# Correction: Lee et al. Tropomyosin-Related Kinase Fusions in Gastrointestinal Stromal Tumors. *Cancers* 2022, *14*, 2659

**DOI:** 10.3390/cancers14225658

**Published:** 2022-11-17

**Authors:** Ji Hyun Lee, Su-Jin Shin, Eun-Ah Choe, Jungyoun Kim, Woo Jin Hyung, Hyo Song Kim, Minkyu Jung, Seung-Hoon Beom, Tae Il Kim, Joong Bae Ahn, Hyun Cheol Chung, Sang Joon Shin

**Affiliations:** 1 Department of Internal Medicine, Division of Medical Oncology, Eunpyeong St. Mary’s Hospital, The Catholic University of Korea, Seoul 03312, Republic of Korea; 2 Department of Pathology, Gangnam Severance Hospital, Yonsei University College of Medicine, Seoul 06273, Republic of Korea; 3 Department of Internal Medicine, Division of Hospital Medicine, Yonsei University College of Medicine, Seoul 03722, Republic of Korea; 4 Department of Medicine, Yonsei University College of Medicine, Seoul 03722, Republic of Korea; 5 Songdang Institute for Cancer Research, Yonsei University College of Medicine, Seoul 03722, Republic of Korea; 6 Department of Surgery, Yonsei University College of Medicine, Seoul 03722, Republic of Korea; 7 Yonsei Cancer Center, Department of Internal Medicine, Division of Medical Oncology, Yonsei University College of Medicine, Seoul 03722, Republic of Korea; 8 Department of Internal Medicine, Institute of Gastroenterology, Yonsei University College of Medicine, Seoul 03722, Republic of Korea

In the original article [1], there was a mistake in Table 1 as published. There were two tumors with tumor size ≤ 5 cm in the *NTRK* fusion group (*n* = 5); however, there were three tumors with tumor size ≤ 5 cm in the five cases in Table 3. Moreover, patients 0 and 1 were low- and intermediate-risk of metastasis, respectively, in Table 1, which was not consistent with the results in Table 3. The corrected Table 1 appears below. Modified numbers are red-colored.

The authors apologize for any inconvenience caused and state that the scientific conclusions are unaffected. The original publication has also been updated.

## Figures and Tables

**Table 1:** Baseline characteristics.

Characteristics	Total (*n* = 31) *N* (%)	*NTRK* Fusion (*n* = 5)	*NTRK* Wild Type (*n* = 26)	*p*-Value
Age				
Median (range)	56 (33–92)	61 (33–92)	53 (37–84)	0.163
Sex	-	-	-	1000
Male	14 (45.2)	2 (40.0)	12 (46.2)	-
Female	17 (54.8)	3 (60.0)	14 (53.8)	-
Tumor size (cm)	-	-	-	-
Median ± SD	8.2 ± 5.3	3.9 ± 6.5	6.0 ± 5.2	0.410
Tumor location	-	-	-	0.028
Abdominal wall	1 (0.3)	0 (0.0)	1 (3.8)	-
Stomach	14 (45.1)	1 (20.0)	14 (53.8)	-
Small bowel	11 (35.4)	2 (40.0)	6 (23.1)	-
Descending colon	1 (0.3)	0 (0.0)	1 (3.8)	-
Rectum	4 (12.9)	2 (40.0)	4 (15.4)	-
Tumor size, groups (cm) ^◇^	-	-	-	1000
≤5	14 (50.0)	3 (60.0)	11 (47.8)	-
>5	14 (50.0)	2 (40.0)	12 (52.2)	--
Mitotic rate ^◐^ (no. of mitoses/50 HPFs *)	-	-	-	0.711
≤5	11 (50.0)	2 (40.0)	12 (52.2)	-
>5	16 (50.0)	3 (60.0)	10 (43.5)	-
Disease status	-	-	-	0.859
No evidence of disease	21 (67.7)	5 (100.0)	16 (61.5)	-
on anticancer treatment	3 (9.7)	0 (0.0)	3 (11.5)	-
Unknown	3 (9.7)	0 (0.0)	3 (11.5)	-
Dead	4 (12.9)	0 (0.0)	4 (15.4)	-
Risk of metastasis ^◈^	-	-	-	0.253
Low	8 (25.8)	1 (20.0)	7 (26.9)	-
Intermediate	3 (9.7)	0 (0.0)	3 (11.5)	-
High	17 (54.8)	4 (80.0)	13 (50.0)	-
Not assessable	3 (9.7)	0 (0.0)	3 (11.5)	-
Surgical treatment	-	-	-	1000
Yes	28 (90.3)	5 (100.0)	23 (88.5)	-
No	3 (9.7)	0 (0.0)	3 (11.5)	-

^◇^ For three inoperable cases, size information and mitotic rate are missing because there was no surgical specimen. ^◐^ Mitotic rate was not evaluated in one surgical specimen. ^◈^ Risk of metastasis was evaluated according to the National Institute of Health (NIH) classification. HPFs: high-power fields; * per 50 HPFs is a total of 5 mm^2^.
