# Supplementary figures and images for: Tropomyosin-Related Kinase Fusions in Gastrointestinal Stromal Tumors
Source: Cancers (Basel). 2022 May 27;14(11):2659. doi: 10.3390/cancers14112659 (PMC9179593; doi:10.3390/cancers14112659)

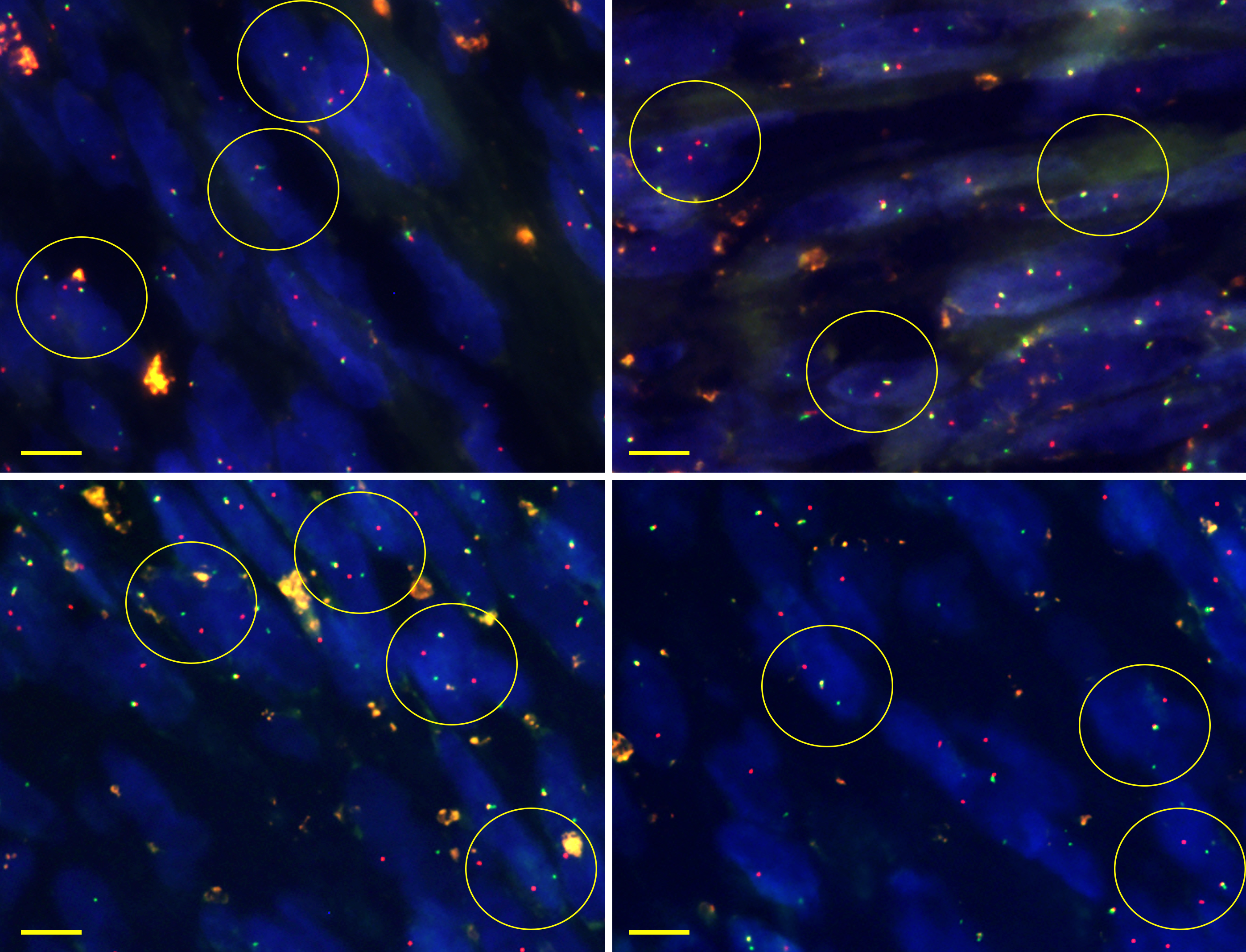

Supplement: Supplementary file 1 [file cancers-14-02659-s001.zip › cancers-1672716-Figure S1.tif]
